# Highly conductive nanometer-thick gold films grown on molybdenum disulfide surfaces for interconnect applications

**DOI:** 10.1038/s41598-020-71520-x

**Published:** 2020-09-02

**Authors:** Yu-Wei Zhang, Bo-Yu Wu, Kuan-Chao Chen, Chao-Hsin Wu, Shih-Yen Lin

**Affiliations:** 1 Graduate Institute of Electronics Engineering, National Taiwan University, No. 1, Sec. 4, Roosevelt Rd., Taipei, 10617 Taiwan; 2 Research Center for Applied Sciences, Academia Sinica, No. 128, Sec. 2, Academia Rd., Taipei, 11529 Taiwan; 3 Department of Photonics, National Cheng Kung University, No. 1 University Road, Tainan City 701, Taiwan

**Keywords:** Electronic properties and materials, Electronic devices

## Abstract

Thin gold (Au) films (10 nm) are deposited on different substrates by using a e-beam deposition system. Compared with sapphire and SiO_2_ surfaces, longer migration length of the Au adatoms is observed on MoS_2_ surfaces, which helps in the formation of a single-crystal Au film on the MoS_2_ surface at 200 °C. The results have demonstrated that with the assistance of van der Waals epitaxy growth mode, single-crystal 3D metals can be grown on 2D material surfaces. With the improved crystalline quality and less significant Au grain coalescence on MoS_2_ surfaces, sheet resistance 2.9 Ω/sq is obtained for the thin 10 nm Au film at 100 °C, which is the lowest value reported in literature. The highly conductive thin metal film is advantageous for the application of backend interconnects for the electronic devices with reduced line widths.

## Introduction

With the rapid shrinkage of devices linewidth to below 3 nm, the short-channel effect and the reduced production yield of silicon (Si) FINFETs have gradually push Si and its device structure to reach the physics limitation in practical application^1–3^. At this stage, there is an urgent need in the semiconductor industry to search for new materials with good device performances in nano-meter scale. In this case, 2D materials have emerged their potential application in the next-generation electronic devices. Since the discovery of the first 2D material, graphene, one of the most interesting phenomenon of these materials is the observation of their unique electrical and optical characteristics in one or few atomic layers^4^. Although high mobility has been demonstrated at its very first publication, however, due to the zero band gap nature of graphene, low ON/OFF ratios are observed for graphene transistors^5,6^. So other 2D materials with bandgaps such as transition metal dichalcogenides (TMDs) has received increasing attention in the last decade^7–9^. Although electronic components made of this material have a high ON/OFF ratio, they exhibit a relatively low carrier mobility than graphene. The limitation of individual 2D materials for device application has again led people to turn their attention to 2D material hetero-structures. In one previous publication, we have demonstrated that with epitaxially grown WS_2_/MoS_2_ hetero-structures, its type-II band alignment would lead to higher drain currents due to the electron injection from the WS_2_ barrier to the MoS_2_ channel^10^. With MoS_2_ as the absorption layer and graphene as the channel layer, epitaxially grown MoS_2_/graphene hetero-structures are also demonstrated for photo-transistor applications^11^.

The demonstration of epitaxially grown vertical 2D material hetero-structures has led to several other investigations of elemental 2D materials grown on molybdenum disulfide (MoS_2_) surfaces^12,13^. Group-V element antimony (Sb) and group-IV elements germanium (Ge) and tin (Sn) have demonstrated their 2D material structures on MoS_2_ surfaces. The results have revealed that through the van der Waals epitaxy growth mode on 2D material surfaces, less dependence to the substrate lattice constants is expected for the epi-layers. Besides 2D materials grown on 2D material surfaces, the other issue arises is the possibility of three-dimensional (3D) crystals grown on 2D material surfaces following the same van der Waals epitaxy on the 2D–3D interfaces. It has been demonstrated in one previous publication that a thin 3D metal layer can be formed on 2D material surface after post-growth annealing^14^. A low contact resistance between the metal/2D material interfaces is also observed in that article. The results have demonstrated that besides 2D material hetero-structures, 3D metals can also be observed on 2D material surfaces. The van der Waals epitaxy taken place on the 2D material surfaces should play an important role for the formation of either 2D or 3D crystals on the 2D material surface. In one previous publication, it has been demonstrated that the strong affinity between gold (Au) and sulfur will lead to large-area and mono-layer MoS_2_ film transferring to different substrates by using the Au-mediated exfoliation method^15^. The same mechanism may also lead to a high sticking coefficient of Au adatoms on MoS_2_ surfaces and form well-stacked Au/MoS_2_ hetero-structures. In this case, the van der Waals epitaxy of Au films with improved crystalline quality could be achieved at relatively low growth temperatures due to the high adatom density on the MoS_2_ surface. Therefore, the investigation of Au film grown on MoS_2_ surfaces should lead to further understanding to the van der Waals epitaxy on 2D material surfaces.

In this paper, 10 nm Au films are deposited on different substrates by using a e-beam deposition system. Compared with sapphire and SiO_2_ surfaces, longer migration length of the Au adatoms is observed on MoS_2_ surfaces. The same mechanism also helps in the formation of a single-crystal Au film on the MoS_2_ surface at 200 °C. The results have demonstrated that with the assistance of van der Waals epitaxy growth mode, single-crystal 3D crystals can be grown on 2D material surfaces. With the improved crystalline quality and less significant Au grain coalescence on MoS_2_ surfaces, close-to-theory sheet resistance 2.9 Ω/sq can be obtained for the thin 10 nm Au film, which is the lowest value reported in literature.

## Results and discussions

### Thin gold films grown on different substrates

**Figure 1:**
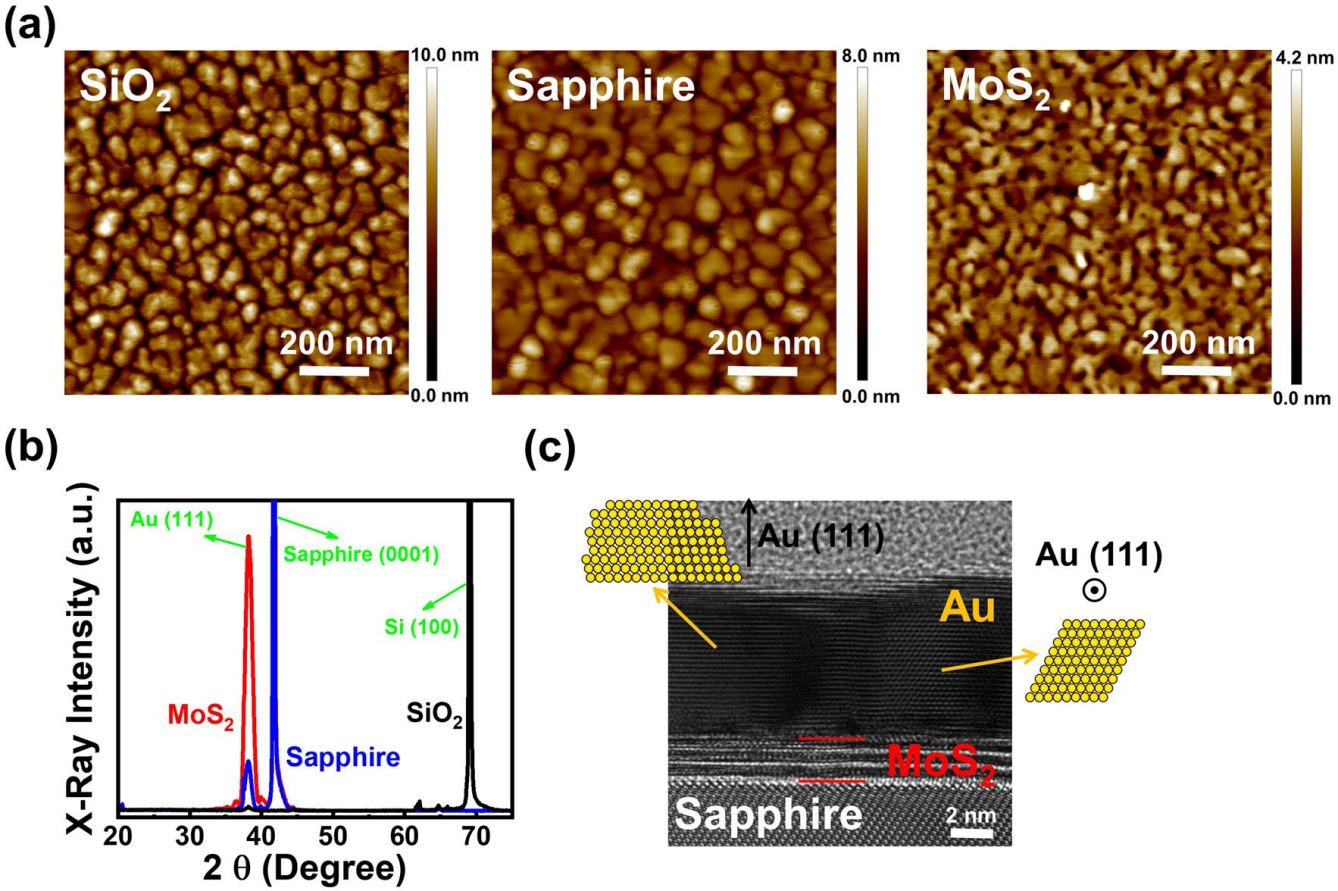
(**a**) 500 × 500 nm^2^ AFM images, (**b**) The XRD curves of three samples with 10 nm Au deposited on 300 nm SiO_2_/Si, c-plane sapphire and tri-layer MoS_2_/c-plane sapphire substrates at RT. (**c**) The cross-sectional HRTEM image of the sample grown on the MoS_2_ surface.

The 500 × 500 nm^2^ atomic force microscopy (AFM) images of 10 nm Au deposited on 300 nm SiO_2_/Si, c-plane sapphire and tri-layer MoS_2_/c-plane sapphire substrates at room temperature (RT) are shown in Fig. 1a. Island growth is clearly observed on the SiO_2_ surface, which may be resulted from the limited Au atom migration due to the numerous dangling bonds on SiO_2_ surfaces. Although larger clusters are observed on sapphire substrates, the thin Au film still exhibits island growth instead of planar film growth. Different with the SiO_2_ and the sapphire surfaces, more continuous film with small holes is observed on the MoS_2_ surface. The results indicate that with no dangling bonds on 2D material surfaces, the migration length of Au atoms is longer than the other two substrate surfaces. In this case, planar film growth instead of island formation would become the preferential growth mode on MoS_2_ surfaces. The 2θ–θ curves of the three samples measured by the X-ray diffraction system (XRD) are shown in Fig. 1b. As shown in the figure, except for the substrate peaks, Au (111) peaks are observed for all the three samples. The most intense Au (111) x-ray peak is observed for the thin Au film grown on the MoS_2_ surface. The results indicate that with the longer adatom migration length, improved crystalline quality can be obtained for the thin Au film on the MoS_2_ surface. To further investigate its crystalline quality, the cross-sectional high-resolution transmission electron microscope (HRTEM) image of the sample grown on the MoS_2_ surface is shown in Fig. 1c. Consistent with the observation from the XRD curve, most of the thin Au film is composed of Au (111) grains along the growth direction (see the supplemental material Fig. S1). However, Au grains with different orientations are still observed in Fig. 1c. The results indicate that although longer adatom migration length and planar Au film growth are observed on the MoS_2_ surface, polycrystalline instead of single-crystal Au film is obtained at room temperature.

### Thin gold films deposited on MoS_2_ surfaces at different growth temperatures

**Figure 2:**
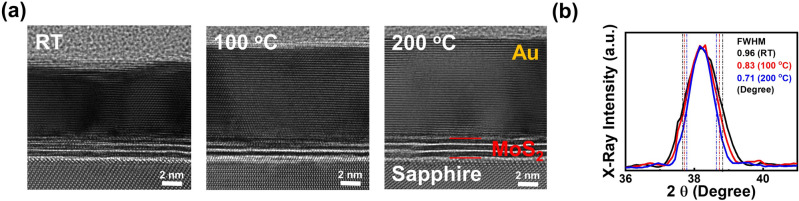
(**a**) The cross-sectional HRTEM images of three samples with 10 nm Au deposited on MoS_2_ surfaces at RT, 100 and 200 °C. (**b**) The normalized Au (111) peaks of the three samples.

To further improve the crystalline quality of the thin Au film, higher growth temperatures are adopted. The cross-sectional HRTEM images of two samples with higher growth temperatures 100 and 200 °C are shown in Fig. 2a. The figure of the sample prepared at RT is shown again in Fig. 2a for comparison. As shown in the figure, increasing Au grain sizes are observed with increasing temperatures. For the sample prepared at 200 °C, the 10 nm Au film exhibits a single crystal orientation as shown in Fig. 2a. The other phenomenon observed in the figure is the increase in film thicknesses with increasing growth temperatures. A possible mechanism responsible for this phenomenon may be the enhanced Au migration length with increasing growth temperatures such that Au grain coalescence and island formation would result in higher Au film thicknesses, which will be discussed in the latter section. The normalized Au (111) peaks of the three samples are shown in Fig. 2b. As shown in the figure, the decreasing full widths at the half maximum (FWHM) of the Au (111) XRD peak from 0.96, 0.83 to 0.71 degree for the three samples prepared at RT, 100 and 200 °C, respectively, are observed. The phenomenon is consistent with the observation of improved crystalline quality with increasing growth temperatures shown in Fig. 2a. As we have discussed in the previous section, with no dangling bonds on 2D material surfaces, the longer migration length of Au atoms on MoS_2_ surfaces would enhance planar growth instead of vertical/island growth of the Au films. In this case, improved crystalline quality and larger single-crystal Au grains would be observed on 2D material surfaces. With increasing growth temperatures, this mechanism will be further enhanced on 2D material surfaces such that even larger single-crystal Au grains are obtained, which will result in decreasing FWHM values of the Au (111) XRD peak as shown in Fig. 2b. However, since the description is focused mostly on the 2D/3D interfaces, further investigation is still required in the future how the initial growth stage would influence the Au films formed on MoS_2_ surfaces. The observations of increasing single-crystal domains and decreasing X-ray peak FWHMs have revealed that with increasing growth temperatures, even longer migration lengths are observed for the Au atoms on MoS_2_ surfaces, which will effectively improve the crystalline quality of the thin Au film. However, although improved crystalline quality is observed with increasing growth temperatures, the possible coalescence of Au grains occur at higher growth temperatures may also result in a more porous film.

### Surface morphologies and growth mechanisms of the thin gold films

**Figure 3:**
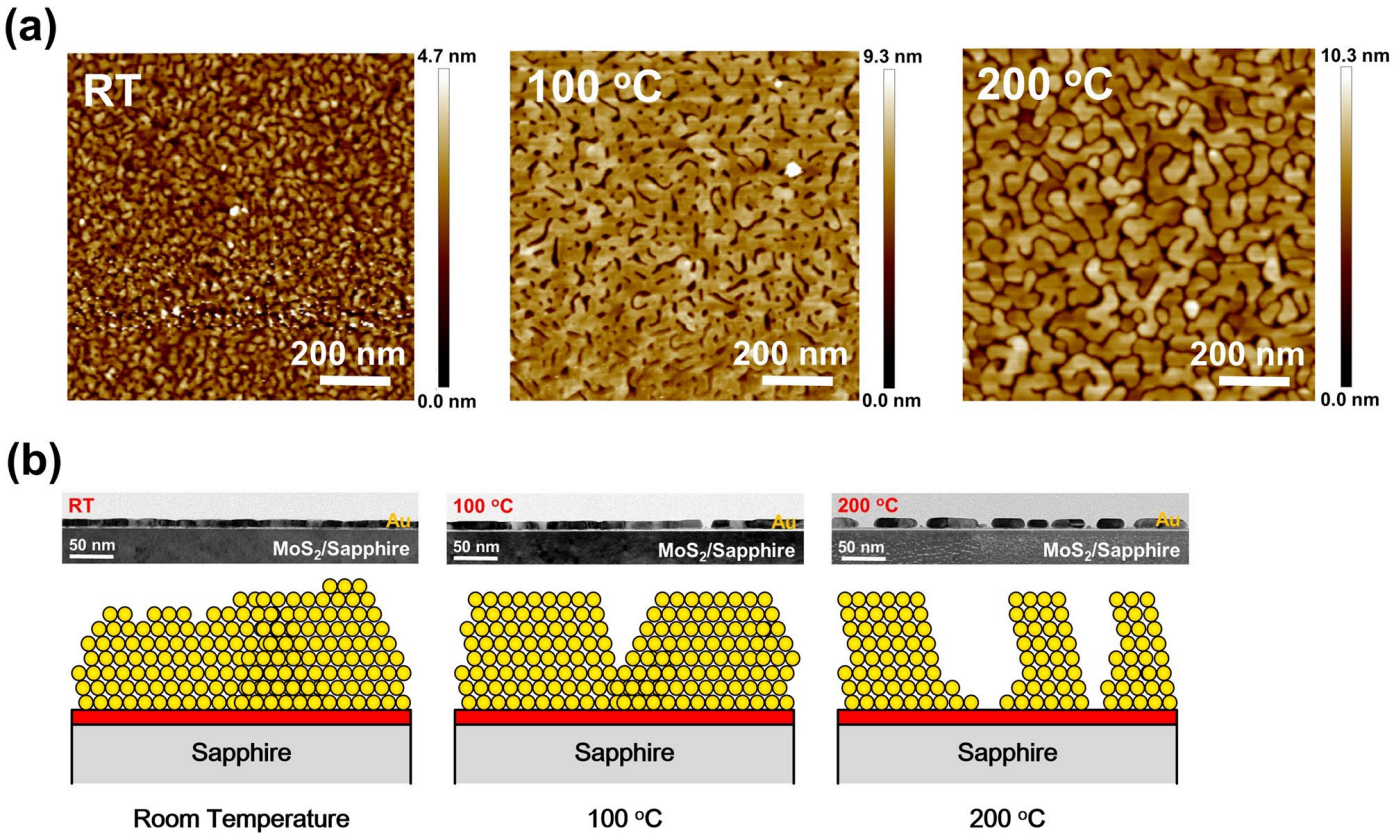
(**a**) 1 × 1 μm^2^ AFM images of three samples with 10 nm Au deposited on MoS_2_ surfaces at RT, 100 and 200 °C. (**b**) The schematic diagrams describing the growth mechanism of the thin Au film. The cross-sectional HRTEM images with lower magnifications of the three samples are also shown in (**b**).

To investigate the morphologies of the thin Au films grown at different temperatures, the 1 × 1 μm^2^ AFM images of the three samples grown at RT, 100 and 200 °C are shown in Fig. 3a. Compared with the sample prepared at RT, the two samples grown at 100 and 200 °C exhibit similar morphologies of porous Au films with connected flat plateaus on MoS_2_ surfaces. With increasing temperature from 100 to 200 °C, the areas of the flat plateaus decrease. A more discontinuous au film is observed for the sample prepared at 200 °C. As extracted from the AFM images, the Au coverage across the wafer still reach 90% for the sample prepared at 100 °C. However, for the sample prepared at 200 °C, the Au coverage drops to 80% across the wafer. In this case, the local discontinuities of the thin Au films may not play a major role on the conductivity of the Au films for the sample grown at 100 °C. A significant reduction in conductivity is expected for the sample prepared at 200 °C, which will be discussed in the latter section. The schematic diagrams describing the growth mechanism of the thin Au film is shown in Fig. 3b. The cross-sectional HRTEM images of the three samples with lower magnifications are also shown in the figure. At room temperature, compared with sapphire and SiO_2_ surfaces, the 2D material surface is of low dangling bond densities. Therefore, the longer migration lengths of the Au adatoms would lead to planar film growth on the MoS_2_ surface instead of island formation on the other two substrate surfaces. In this case, a continuous Au film is observed on the HRTEM image. With increasing growth temperature to 100 °C, the further extended adatom migration lengths would lead to improved crystalline quality and the appearance of flat Au (111) surfaces. In this case, flat Au plateaus would appear on MoS_2_ surfaces. Although local discontinuity is observed, relative continuous film is still observed for the thin Au film grown at 100 °C. With an even higher growth temperature up to 200 °C, the significant adatom migration would lead to improved crystalline quality as shown in Fig. 2a. However, the severe Au grain coalescence would result in a more discontinuous film as shown in the HRTEM image at Fig. 3b. On the other hand, although more discontinuous film is observed for thin Au films, the improved crystalline quality of Au films at high growth temperatures on MoS_2_ surfaces may lead to a flat large-area single-crystal Au (111) film growth on 2D material surfaces when thicker Au films are prepared (see the supplemental material Fig. S2). The thick single-crystal metal film growth on 2D material surfaces may have other applications in surface sciences and is also a result of van der Waals epitaxy occurred on the 2D–3D interface. Besides the van der Waals epitaxy growth mode on 2D material surfaces, the crystal symmetry of the MoS_2_ film may also influence the grain size of the Au films. Further investigation is still required to clarify the detailed growth mechanisms of Au films on MoS_2_ surfaces.

### Applications of conductive nanometer-thick gold films

As we have discussed in the introduction section, with the shrinkage of device sizes, 2D material can be applied for the next-generation electronic devices. On the other hand, the decreasing metal film thicknesses will also bring in significant increase in resistance for interconnects. In this case, the superior crystalline quality of the thin Au films grown on 2D material surfaces may help to reduce the resistance of the thin interconnects. The sheet resistance values of the 10 nm Au films grown at room temperature on MoS_2_ and sapphire surfaces are 9.03 and 5.47 × 10^2^ Ω/sq, respectively. Open circuit is observed for the thin Au film deposited on the SiO_2_ surface. As discussed in the previous section, the results demonstrate that the longer migration length of Au adatoms on the 2D material surface does help in planar film growth and therefore, lower sheet resistance can be obtained on the MoS_2_ surface. The sheet resistance can be further reduced down to 2.90 Ω/sq for the thin Au film grown on the MoS_2_ surface at 100 °C. Since the resistivity of gold is 2.45 × 10^−8^ Ω m, the sheet resistance value 2.90 Ω/sq is actually close to the theoretical limit 2.45 Ω/sq of the 10 nm gold film (sheet resistance = resistivity/film thickness for thin metal films). For the sample prepared at 200 °C, the sheet resistance of the 10 nm Au film increases to 3.72 × 10^3^ Ω/sq, which is consistent with the previous observation that a more discontinuous film will be obtained due to the Au grain coalescence at higher growth temperatures. The results have demonstrated that with the van der Waals epitaxy of Au films on 2D material surfaces, preferential planar film growth resulted from the longer migration lengths of Au adatoms would help to increase the conductivity of the thin metal film. By increasing growth temperatures with limited grain coalescence, further reduced sheet resistance value can be observed for the thin metal film due to the improved crystalline quality.

**Figure 4:**
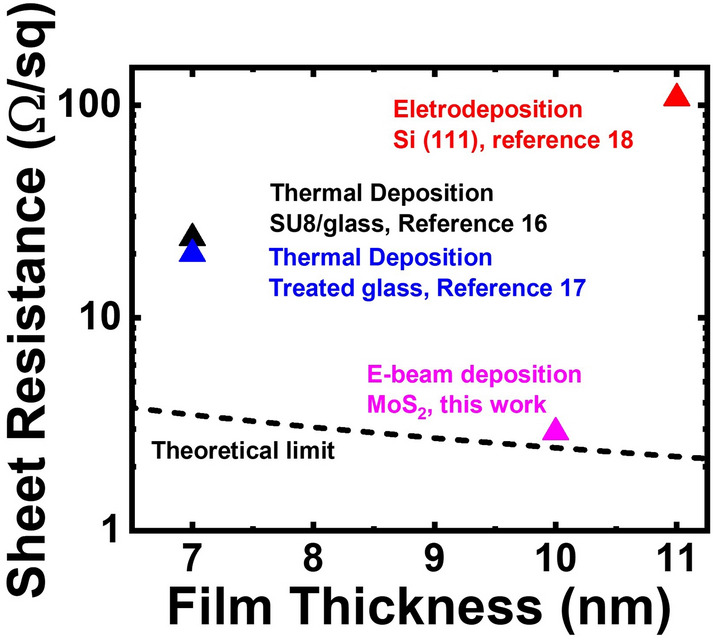
The sheet resistance values of thin Au films prepared by using different approaches and subtracts in literature. The sheet resistance value of the 10 nm Au film deposited on the MoS_2_ surface at 100 °C is also shown in the figure. The dashed curve shows the theoretical limit for the sheet resistance of thin Au films (sheet resistance = resistivity/film thickness).

In previous publications, different approaches have been adopted for the fabrication of conductive thin Au films^16–18^. The sheet resistance values of the Au films with ~ 10 nm thicknesses reported in literature are shown in Fig. 4. The sheet resistance value of the 10 nm Au film deposited on the MoS_2_ surface at 100 °C observed in this report is also shown in the figure. By using the thermal evaporation and treated glass substrates, low sheet resistance values 23.75 and 20 Ω/sq are reported for 7 nm Au films^16,17^. By using the electrodeposition, it has been reported that single-crystal Au films can be obtained on Si (111) substrates. However, a higher sheet resistance 369 Ω/sq is reported for the 7 nm Au film^18^. Slightly lower sheet resistance 108 Ω/sq is for the sample with the 11 nm Au film^18^. Compared with the values 20–100 Ω/sq obtained for thin Au films ~ 10 nm reported in literature, low sheet resistance 2.90 Ω/sq for the sample grown on the MoS_2_ surface at 100 °C is observed in this report. To our knowledge, this is the lowest sheet resistance value reported in literature.

## Conclusion

In conclusion, we have demonstrated that with the assistance of van der Waals epitaxy growth mode on 2D material surfaces, single-crystal Au films can be grown on MoS_2_ surfaces at low growth temperature 200 °C. With the improved crystalline quality and the preferential planar film growth on MoS_2_ surfaces, close-to-theory sheet resistance can be obtained for the thin 10 nm Au film. The results have demonstrated that with the van der Waals epitaxy of 3D crystals on 2D material surfaces, preferential planar film growth resulted from the longer migration lengths of Au adatoms would help to increase the conductivity of the thin metal film. Further investigation is still required on how the initial growth stage on the 2D/3D interface would influence the Au films formed on MoS_2_ surfaces. By increasing growth temperatures with limited grain coalescence, further reduced sheet resistance value can be observed for the thin metal film due to the improved crystalline quality. The highly conductive thin gold films can be advantageous for the application of backend interconnects.

## Methods

Three different substrates including (a) c-plane sapphire, (b) 300 nm SiO_2_/Si and tri-layer MoS_2_ on c-plane sapphire substrates are used for the thin Au film deposition. For the growth of tri-layer MoS_2_, the Mo metal was deposited on the sapphire substrates by using a radio-frequency (RF) sputtering system. The sputtering power was kept at 30 W, and the background pressure was maintained at 5 × 10^−3^ Torr with a 40 sccm gas flow of argon (Ar). The sputtering time is 45 s for the preparations of MoS_2_. After the metal deposition, the sample was placed at the center of the hot furnace for sulfurization. There was used 160 sccm Ar gas as carrier gas while the pressure was kept at 0.7 Torr. The growth temperature of the sample was kept at 800 °C with 1.5 g of sulfur(S) powder placed upstream of the gas flow^19^. The layer numbers of the MoS_2_ film are determined by using the atomic layer etching technique (ALE)^20,21^. The Raman spectra of the tri-layer MoS_2_ after repeated ALE procedures are shown in the supplemental material Fig. S3. The 10 nm Au films are deposited on different substrates by using a e-beam deposition system. Before the metal deposition, the chamber pressure was pumped down to 3 × 10^−7^ torr by using a mechanical pump followed by a cryo-pump. The deposition rate of the Au film is kept at 0.1 Å per second. The deposition rate and the thickness of the thin Au films quartz are determined by a crystal microbalance (QCM) equipped with the e-beam system. For the four-point probe measurements, a four-pin probe station KSR-4 with BeCu probe tip and tip pitch 1 mm is adopted. Keithley 2,400 SourceMeter is used to measure the sheet resistance of the samples. The cross-sectional HRTEM images measurement are obtained by using a JEOL JEM-2800F transmission electron microscopy system operated at 200 kV. The XRD measurement are done by using a Bruker New D8 Discover XRD system. The atomic force microscopy (AFM) measurement are done by using a BRUKER Dimension ICON AFM system.

## Supplementary information

Supplementary information
