# Supplementary material for: Highly conductive nanometer-thick gold films grown on molybdenum disulfide surfaces for interconnect applications
Source: Sci Rep. 2020 Sep 2;10:14463. doi: 10.1038/s41598-020-71520-x (PMC7468125; doi:10.1038/s41598-020-71520-x)
Supplement: Supplementary file 1 — Supplementary information [file 41598_2020_71520_MOESM1_ESM.pdf]

Highly Conductive Nanometer-Thick Gold Films Grown on Molybdenum Disulfide Surfaces for Interconnect Applications

Yu-Wei Zhang[1, 2], Bo-Yu Wu[3], Kuan-Chao Chen[2], Chao-Hsin Wu[1], and Shih-Yen Lin[1, 2, 3 *]

[1]Graduate Institute of Electronics Engineering, National Taiwan University, No. 1, Sec. 4, Roosevelt Rd., Taipei 10617, Taiwan

[2]Research Center for Applied Sciences, Academia Sinica, No. 128, Sec. 2, Academia Rd., Taipei 11529, Taiwan

[3]Department of Photonics, National Cheng Kung University, No. 1 University Road, Tainan City 701, Taiwan

*Corresponding author, electronic mail: shihyen@gate.sinica.edu.tw

The cross-sectional HRTEM image with lower magnification of the 10 nm Au film deposited on the MoS_2 surface at RT is shown below. As shown in the figure, most of the thin Au film is composed of Au (111) grains along the growth direction, which is consistent with the observation of intense Au (111) peak from the XRD curve.

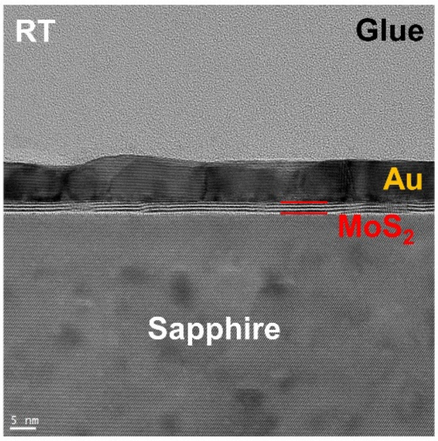

Figure S1. The cross-sectional HRTEM image with lower magnification of the 10 nm Au film deposited on the MoS_2 surface at RT.

The scanning electron microscopy (SEM) image of a thick Au film (50 nm) deposited on a tri-layer MoS_2/sapphire substrate at 400 °C. As shown in the figure, a flat and single-crystal Au film is obtained on the MoS_2 surface. Only scattered small holes are observed on the Au film. The results demonstrate that with improved crystalline quality at high growth temperatures, single-crystal 3D crystals with flat surfaces can be obtained on 2D material surfaces. The van der Waals epitaxy occurred on the 2D-3D interface plays an important role for the formation of high-quality 3D crystals on 2D material surfaces.

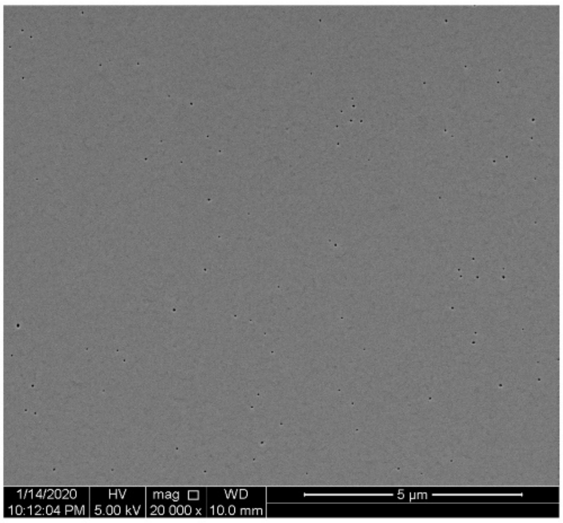

Figure S2. The SEM image of a thick Au film (50 nm) deposited on a tri-layer MoS_2/sapphire substrate at 400 °C by using the e-beam deposition system.

The pictures of the MoS_2 film after each atomic-layer etching (ALE) procedure are shown below. The corresponding Raman spectra are also shown below. As shown in the figure, after ALE procedure, the Δk value would decrease, which indicates the decrease in the MoS_2 layer

numbers. After three ALE procedures, the color of the sample is close to blank sapphire substrates and no characteristic Raman peaks of MoS_2 are observed for the sample. The results suggest a tri-layer MoS_2 is grown on the sapphire substrate.

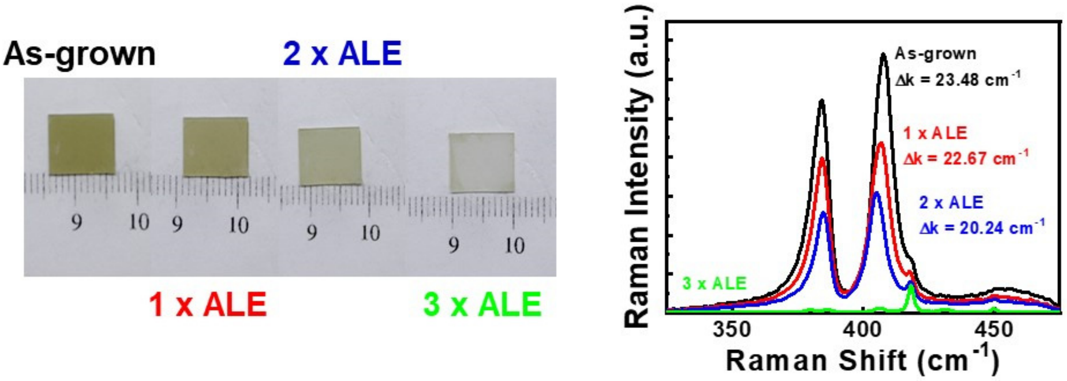

Figure S3 The pictures of the MoS_2 film after each ALE procedure (left) and their corresponding Raman spectra (right).
